# Supplementary material for: An embryonic stem cell-specific heterochromatin state promotes core histone exchange in the absence of DNA accessibility
Source: Nat Commun. 2020 Oct 9;11:5095. doi: 10.1038/s41467-020-18863-1 (PMC7547087; doi:10.1038/s41467-020-18863-1)

An embryonic stem cell-specific heterochromatin state promotes core histone exchange in the absence of DNA accessibility

Navarro et. al.

Supplementary Information

Supplementary Figure 1

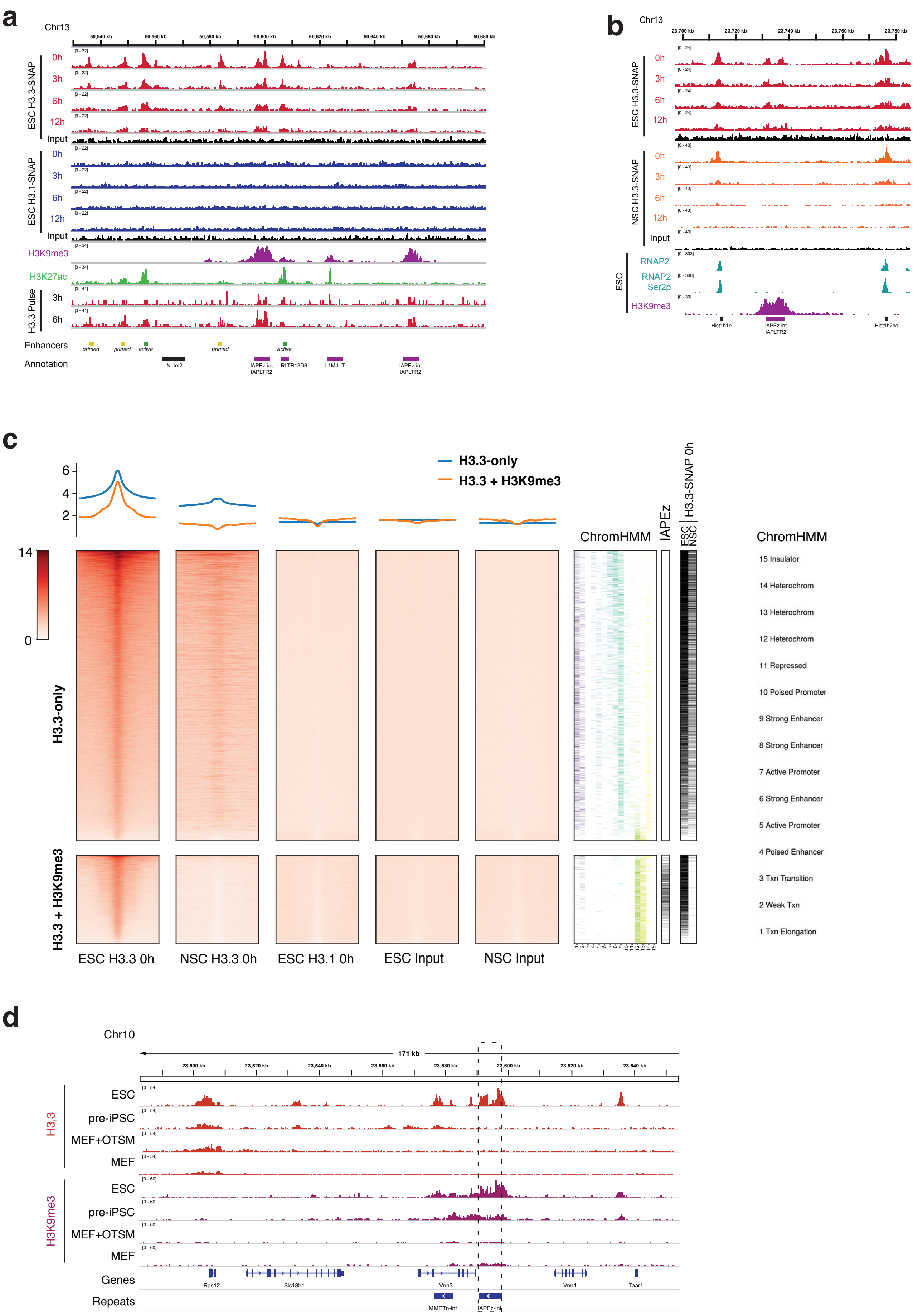

Supplementary Figure 1 - related to Figure 1a, b - Additional examples and controls of H3.3 enrichment at active regions and interstitial heterochromatin.

a Genome tracks example of time-ChIP data (H3.3-SNAP and H3.1-SNAP timepoints 0h throughout 12h) in mouse embryonic stem cells (ESC), together with H3.3-HA pulse data [1,2], H3K27ac and H3K9me3 [3,4]. H3.3-SNAP and H3K27ac enriched regions show overlap with active and primed enhancers. H3.3-SNAP and H3K9me3 coincide with IAP ERV (see also genome-wide analysis in Supplementary Figure 3). H3.1-SNAP and Input controls show no particular enrichment. All tracks were normalized to Reads Per Genomic Content (RPGC). **b** Genome tracks example showing time-ChIP H3.3 data in both mouse embryonic stem cells (ESC) and neural stem cells (NSC), together with H3K9me3 ChIP [3] and RNA Polymerase II ChIP [5] at Hist1 histone gene cluster. H3.3 enrichment and turnover at IAP elements is observed in ESC but not NSC, whereas H3.3 enrichment and turnover at the housekeeping genes is maintained in NSC. **c** Density heatmaps and average profiles (RPGC) of time-ChIP H3.3-SNAP in embryonic stem cells (ESC) and neural stem cells (NSC) and H3.1-SNAP at timepoint 0h in ESC over endogenous H3.3 peaks in ESC coinciding (H3.3+H3K9me3) or not (H3.3-only) with H3K9me3 peaks [6]. Overlap with 15-State ChromHMM annotation [7] states, IAP ERV elements (IAPEz-int and flanking LTRs from mm9 RepeatMasker track), and H3.3-SNAP peaks called in ESC and NSC is shown. Additionally, ESC and NSC input data is also shown. **d** H3.3 and H3K9me3 profiles along a reprogramming time-course from mouse embryonic fibroblasts (MEF) [8]. Early reprogramming intermediate (48h after expression of reprogramming factors Oct4, Nanog, Klf4, Myc) and late reprogramming intermediate stable cell line (pre-iPSC) are compared to MEF and ESC. All tracks are plotted on the same scale as RPGC. H3K9me3 is first enriched at IAP ERV at the pre-iPSC intermediate and further increases in ESC. H3.3 is exclusively acquired at IAP ERVs in ESC, while nearby housekeeping gene *Rps12* is enriched consistently in all cell types.

Supplementary Figure 2

a

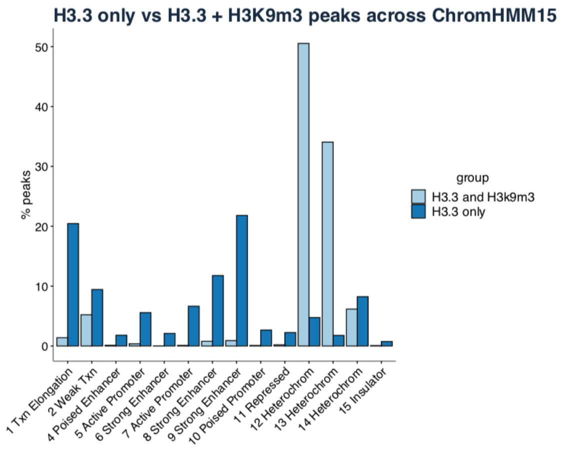

Supplementary Figure 2 - related to Figure 1d - Annotation of H3.3 peaks using 15-State ChromHMM model

Barplot showing relative overlap of H3.3-only and H3.3+H3K9me3 peak sets [6] with 15 State ChromHMM annotation, grouped by coincidence with H3K9me3 peaks. Each peak is assigned the ChromHMM15 state that overlaps with it the most. Amounts are shown as a percentage of the total peak set. H3.3-only peaks overlap the most with strong enhancers and transcription elongation states, whereas H3.3+H3K9me3 peaks overlap with heterochromatin states 12-14, defined by absence of H3K4me3, H3K4me1, H3K27ac, H3K36me3 and RNAP2 marks.

Supplementary Figure 3

Genome-wide analysis in ~545k 5kb bins (Log2 fold-enrichment over input)

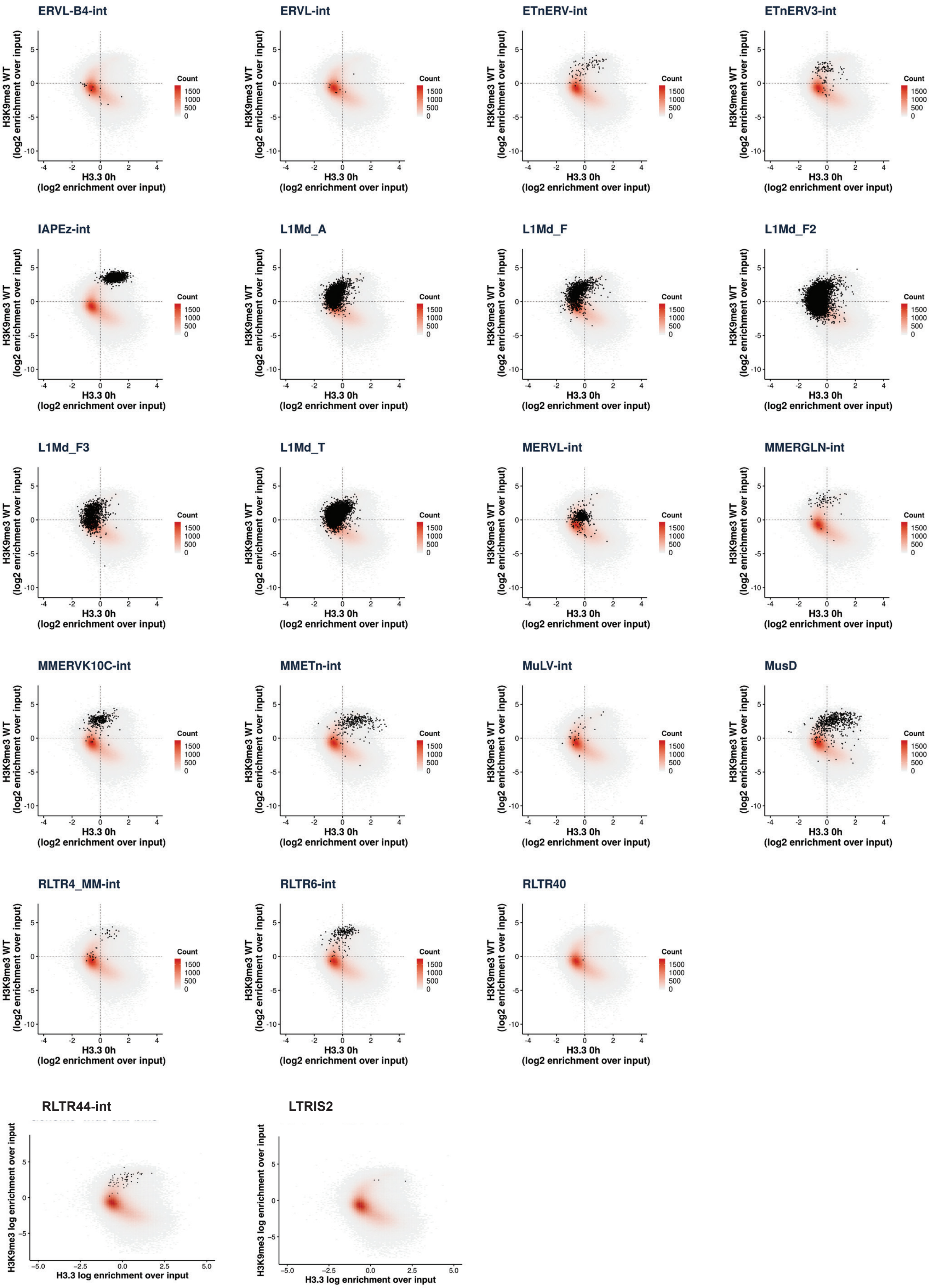

Supplementary Figure 3 - related to Figure 1e - Genome-wide correlation of H3.3 and H3K9me3 is shaped by a subset of ERV families

Scatter plot showing relationship between H3.3 and H3K9me3 signal assessed over 5kb bins genome-wide. Genome was partitioned in 5kb bins and coverage enrichment was calculated as log2 fold change of each signal over their corresponding input. Color of each tile represents point density of all the bins with similar H3K9me3 and H3.3 values (on a tile-resolution of 0.1). RepMasker annotation was intersected with 5kb bins for major repeat families. Bins that overlap with the corresponding repeat type are shown in black. In the rare case where more than one element overlapped with the same bin, the repeat type that overlapped the most was adjudicated.

Supplementary Figure 4

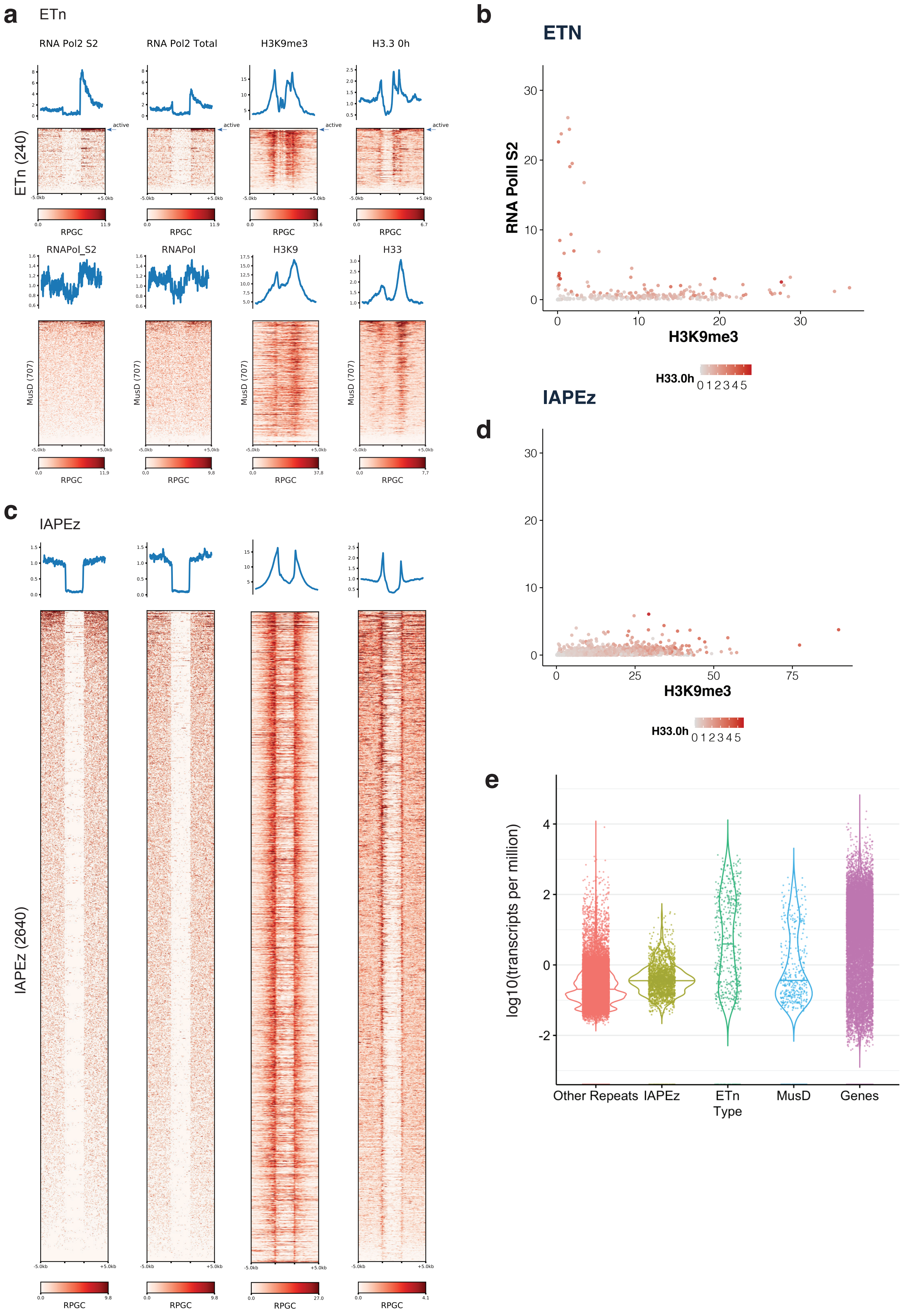

Supplementary Figure 4 - related to Figure 1e - ETn but not IAP ERV families contain a small number of active elements in the annotated genome. a Heatmap showing RNA Polymerase II and H3K9me3 signal [3,5] over unique 5kb flanking regions of ETn and MusD repeat elements. Among the annotated ETn elements, a small subset (marked with a blue arrow) show high RNA Polymerase II signal at their 3' flanking region together with absence of H3K9me3 , which suggests that these individual elements are transcribed at high levels, whereas the remaining elements are in a repressed or inactive state. **b** Scatterplot showing enrichment of RNA Pol II CTD Serine 2 phosphorylation versus H3K9me3 [3,5] over ETn elements and 1kb flanking regions, using only uniquely mappable reads. H3.3 enrichment is overlaid as colorscale. ~10 elements appear actively transcribed and lack H3K9me3, enrichment of histone H3.3 in these cases may stem from transcription associated nucleosome turnover. **c** Heatmap showing RNA Polymerase II and H3K9me3 signal over 5kb flanking regions of annotated IAP elements. No actively transcribed IAP ERVs are discernable by RNA Polymerase II signal. **d** Scatterplot showing enrichment of RNA Pol II CTD Serine 2 phosphorylation versus H3K9me3 [3,5] over IAP elements and 1kb flanking regions, using only uniquely mappable reads. H3.3 enrichment is overlaid as colorscale. **e** Violin plot of RNA-Seq transcript levels for genes and selected repeat elements. RNA-Seq read counts were mapped to mm9 and read counts were extracted over RefSeq genes and RepMasker annotation. Read counts were normalized to the total base pairs covered by each gene or repeat instance (RPK, reads per kilobase) and scaled to transcripts per kilobase million (TPM) and plotted as in log scale.

Supplementary Figure 5

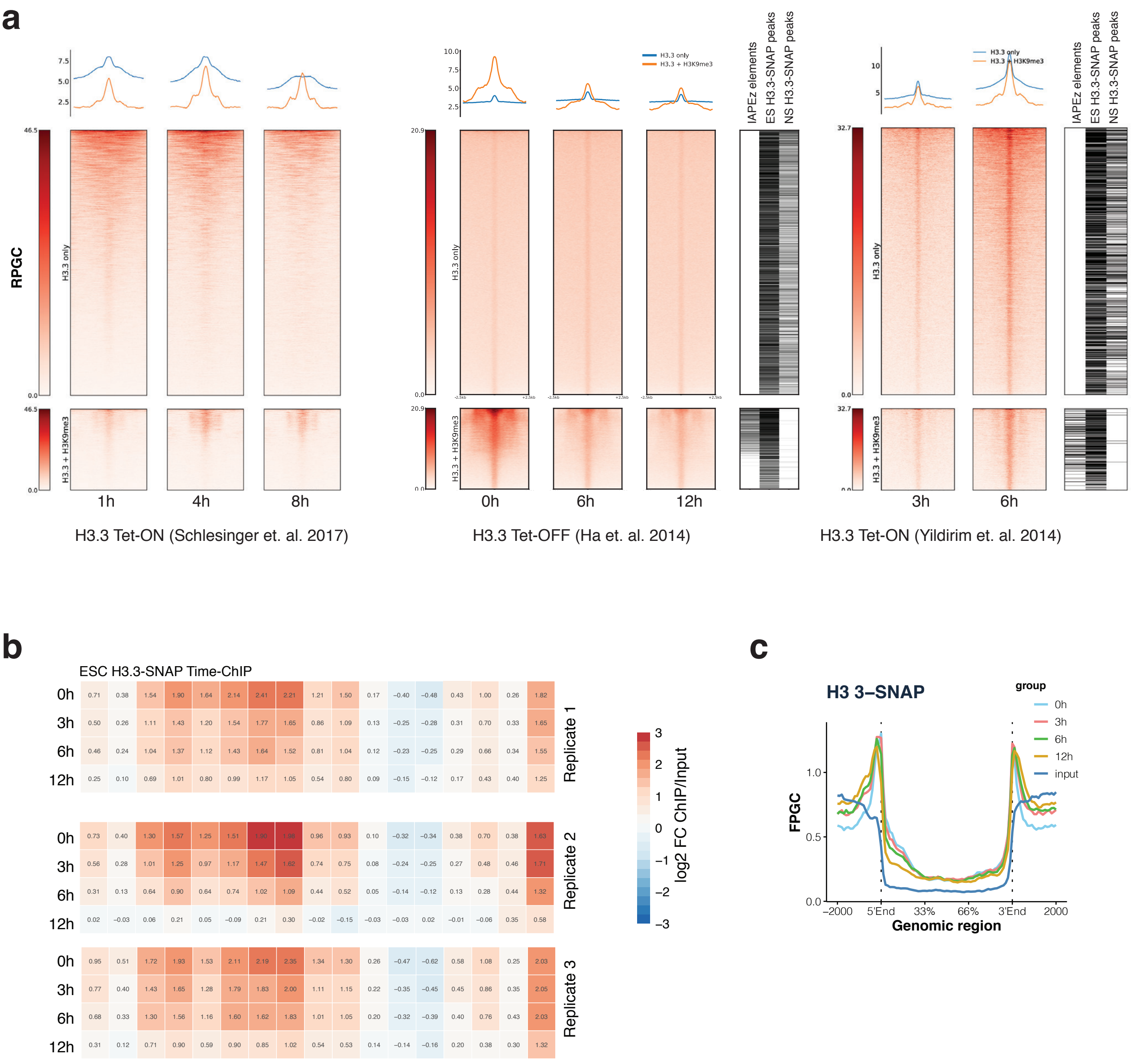

Supplementary Figure 5 - related to Figure 2a-e - Analysis of additional H3.3 pulsing datasets. **a** Peak Density Heatmaps and average profiles of H3.3 pulse or chase data from three different studies in mouse ESC [1,9,10] at H3.3-only and H3.3+H3K9me3 peak sets [6]. **b** Mean read density heatmap showing enrichments of H3.3-SNAP time-ChIP (log2 fold-change over input) over 15 ChromHMM states as well as H3K9me3 and H3.3+H3K9me3 enriched regions [6]. Same as Figure 2d but with additional replicates [2]. **c** Average coverage of H3.3-SNAP time-ChIP uniquely mappable reads over 2640 shared IAP ERVs. Fragments defined by paired-end reads were piled up and normalized by 1x Genome coverage (Fragments Per Genomic Content, FPGC)

Supplementary Figure 6

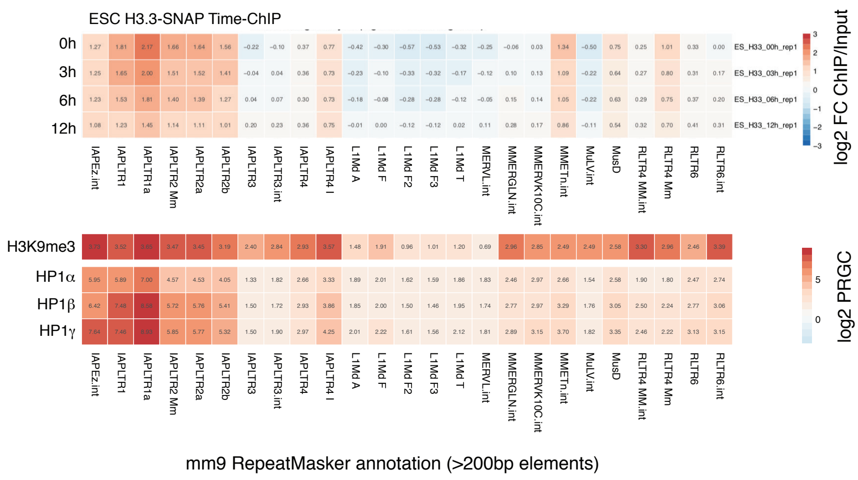

Supplementary Figure 6 - related to Figure 2d - Analysis of repetitive element families

a Mean read density heatmaps of time-ChIP H3.3-SNAP data at a major repetitive elements from RepeatMasker annotation (filtered for elements larger than 200bp). Each category enrichment is calculated as log2 fold change of mean coverage over input. IAPLTR1a shows the highest enrichment at time point 0h and turnover. Most IAP LTRs and internal sequences, as well as ETn and MusD elements show both enrichment and H3.3 turnover.

Extended Data Figure 7

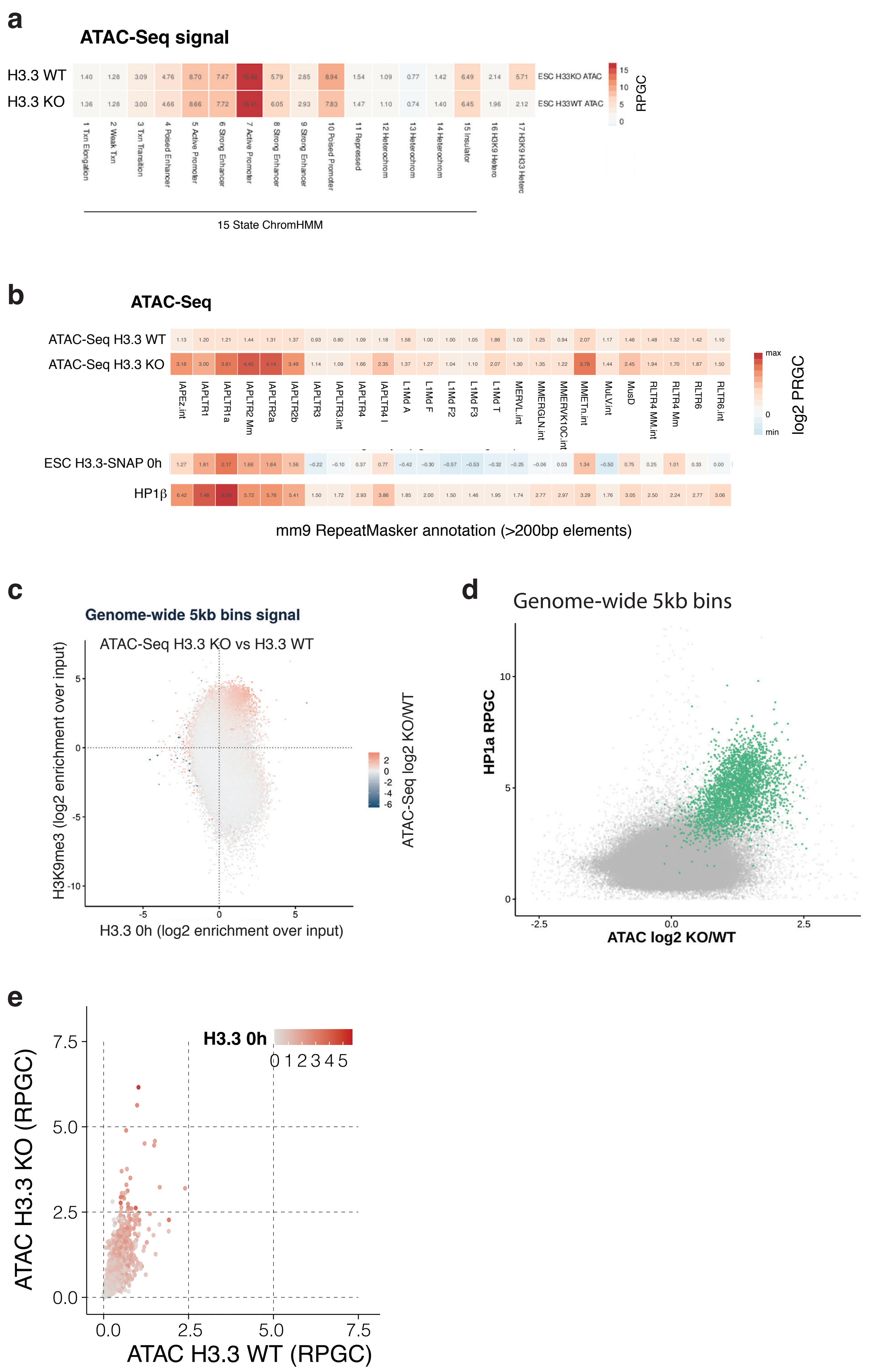

Supplementary Figure 7 - related to Figure 3a, d - Genome-wide maintenance of accessibility in the absence of H3.3KO cells independent of active or inactive chromatin state, and selective gain in accessibility at H3K9me3-H3.3 co-enriched regions.

a Mean read density heatmap of ATAC-seq data of H3.3 wildtype and knockout cells [4]. Both WT and KO show very similar genome-wide read density levels across all 15 ChromHMM States. While H3K9me3-only regions remain inaccessible, a striking ~3-fold increase in ATAC-Seq signal is found at H3.3 + H3K9me3 regions in H3.3KO cells. **b** Mean read density heatmap of ATAC-seq data of H3.3 wildtype and knockout by repeat family. Increase in accessibility is observed for those repeat families that are enriched in H3.3 in wildtype cells. **c** Tile-plot showing relationship between H3.3 and H3K9me3 signal assessed over 5kb bins genome-wide. Each tile color shows log2 fold enrichment of average ATAC-seq signal for H3.3 KO over WT of all the bins with similar H3K9me3 and H3.3 values (on a tile-resolution of 0.1). A majority of zero values show ATAC-seq signal is very similar genome-wide, with H3.3 KO showing highest enrichment over WT on regions both H3.3- and H3K9me3-enriched. **d** Scatter plot of 5kb bins, showing HP1a occupancy [11] versus log2-fold change in ATAC-Seq signal upon H3.3 knockout. Bins overlapping with IAP ERVs are drawn in green. **e** Scatter plot showing uniquely mappable ATAC-Seq reads (RPGC) in H3.3 KO versus WT cell lines, at 2640 shared IAP ERVs and 1kb flanking regions. H3.3-SNAP 0h density by unique reads is overlaid as color scale.

Supplementary Figure 8

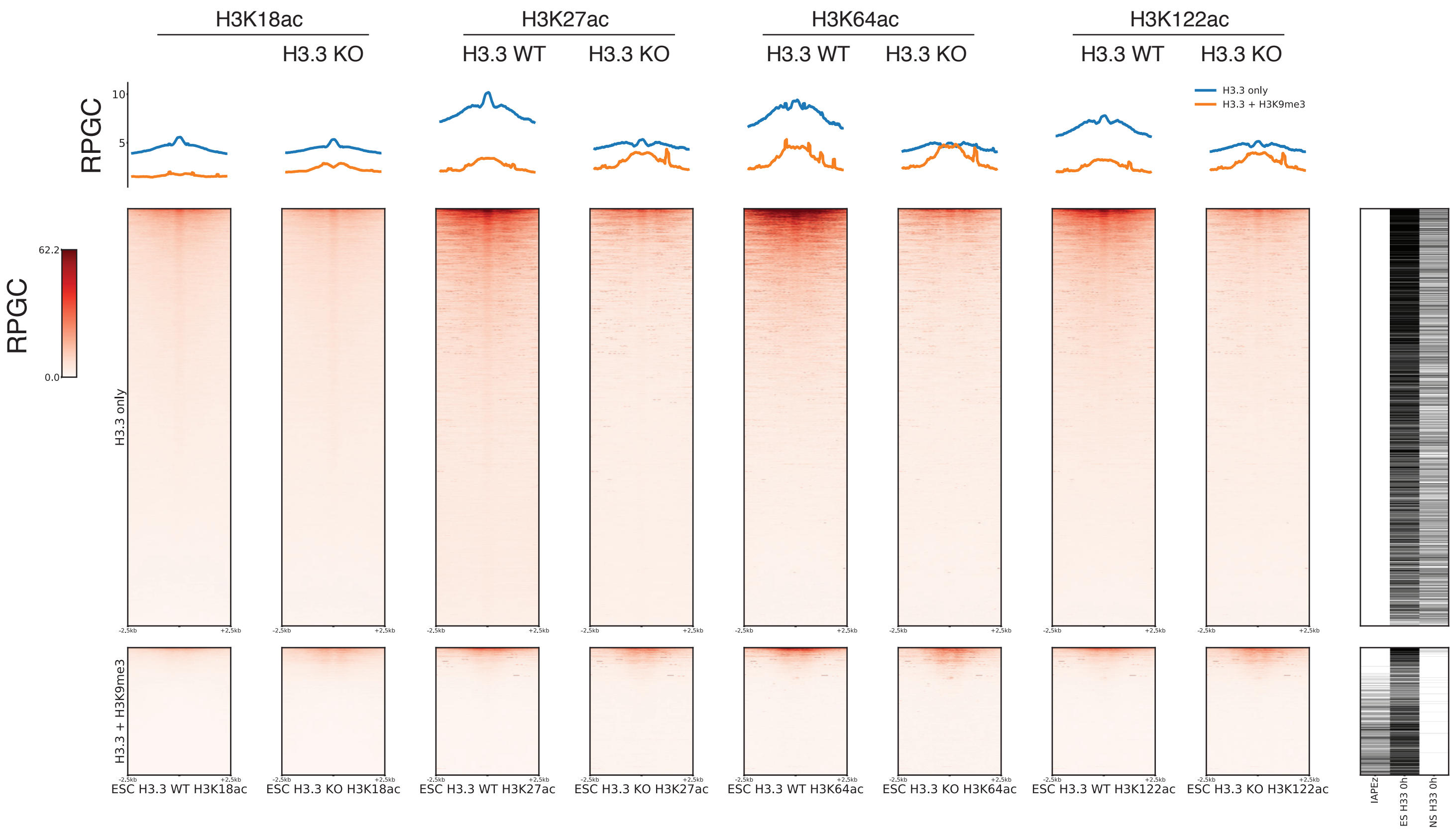

Supplementary Figure 8 - related to Figure 3a - Loss of H3.3 does not lead to an increase in histone acetylations at H3.3+H3K9me3 regions.

a Density Heatmaps and average profiles of H3K18ac, H3K27ac, H3K64ac and H3K122ac marks for H3.3 WT and KO samples [4] at H3.3-only and H3.3+H3K9me3 peak sets. As previously described, histone acetylation, present at a subset of H3.3 peaks, is reduced in the absence of H3.3 [4], but basal levels at H3.3+H3K9m3 regions are not changed.

Supplementary Figure 9

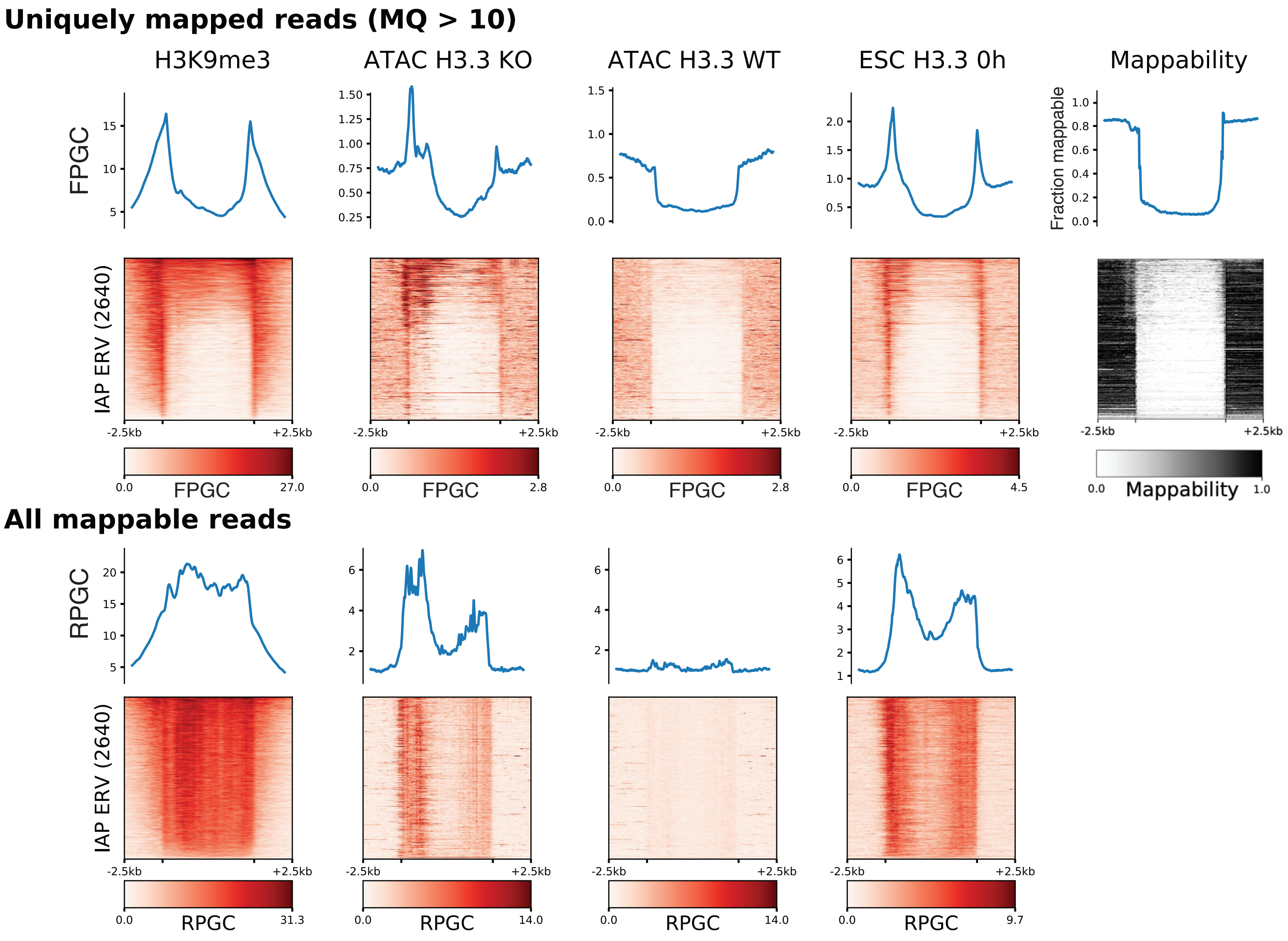

Supplementary Figure 9 - related to Figure 3f - Uniquely mappable reads show widespread increase in DNA accessibility over individual IAP ERV elements. Density heatmap of uniquely mappable paired-end reads within and adjacent to 2640 shared IAP ERVs for H3K9me3, H3.3 KO and WT ATAC-seq, and Time-ChIP H3.3-SNAP at timepoint 0h. Top row shows average profiles. Uniquely mapped reads at flanking regions allow to distinguish among individual instances of repetitive elements, showing that the observed effect is pervasive across such repeat elements. Lower row shows the corresponding unfiltered signal including multi-mappable reads for the same datasets at the same IAP ERVs set. Multimappable reads were assigned randomly to one of the ambiguous matches. On the rightmost column, a 100bp mappability track that flanking regions and some internal sections of IAP ERVs are uniquely mappable.

Supplementary Figure 10

a

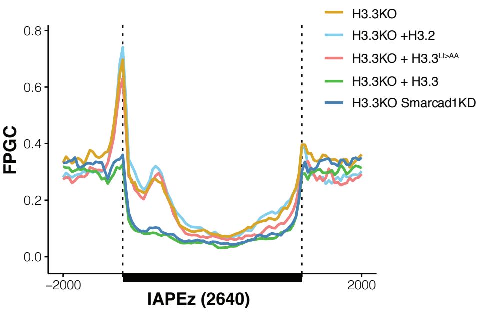

b

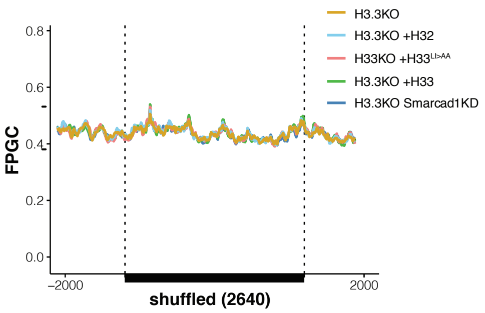

Supplementary Figure 10 - related to Fig 4c, d - ATAC-Seq signal at IAP ERVs and unique genomic regions. a Average coverage of ATAC-Seq signal from uniquely mappable fragments over 2640 shared IAP ERVs including data from Martire et. al. [4], and this study. Fragments defined by paired-end reads were piled up and normalized to 1x Genome coverage (Fragments Per Genomic Content, FPGC). ATAC-Seq coverage in H3.3 KO is compared with H3.3 WT, and H3.3 KO complemented with wildtype H3.3, H3.2 or $H3.3^{LI>AA}$. **b** Control for a) where IAP ERV genomic intervals were randomly shuffled across the mm9 genome, creating a control group of the same number and length.

Supplementary Figure 11

a ATAC-Seq

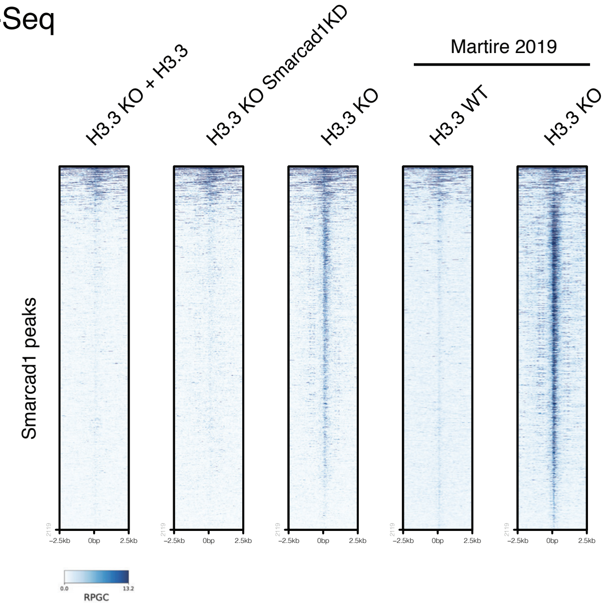

Supplementary Figure 11 - related to Fig 5d - ATAC-Seq signal at Smarcad1 binding sites. ATAC-Seq and ChIP-Seq read density heatmaps and average profiles over 2670 Smarcad1 peaks. ATAC-Seq coverage in H3.3 KO cells and H3.3 KO cells complemented with wildtype H3.3, H3.2 or H3.3LI>AA from this study is shown next to ATAC-Seq from H3.3 WT and the same H3.3 KO cells from Martire et. al. [4].

Supplementary Figure 12

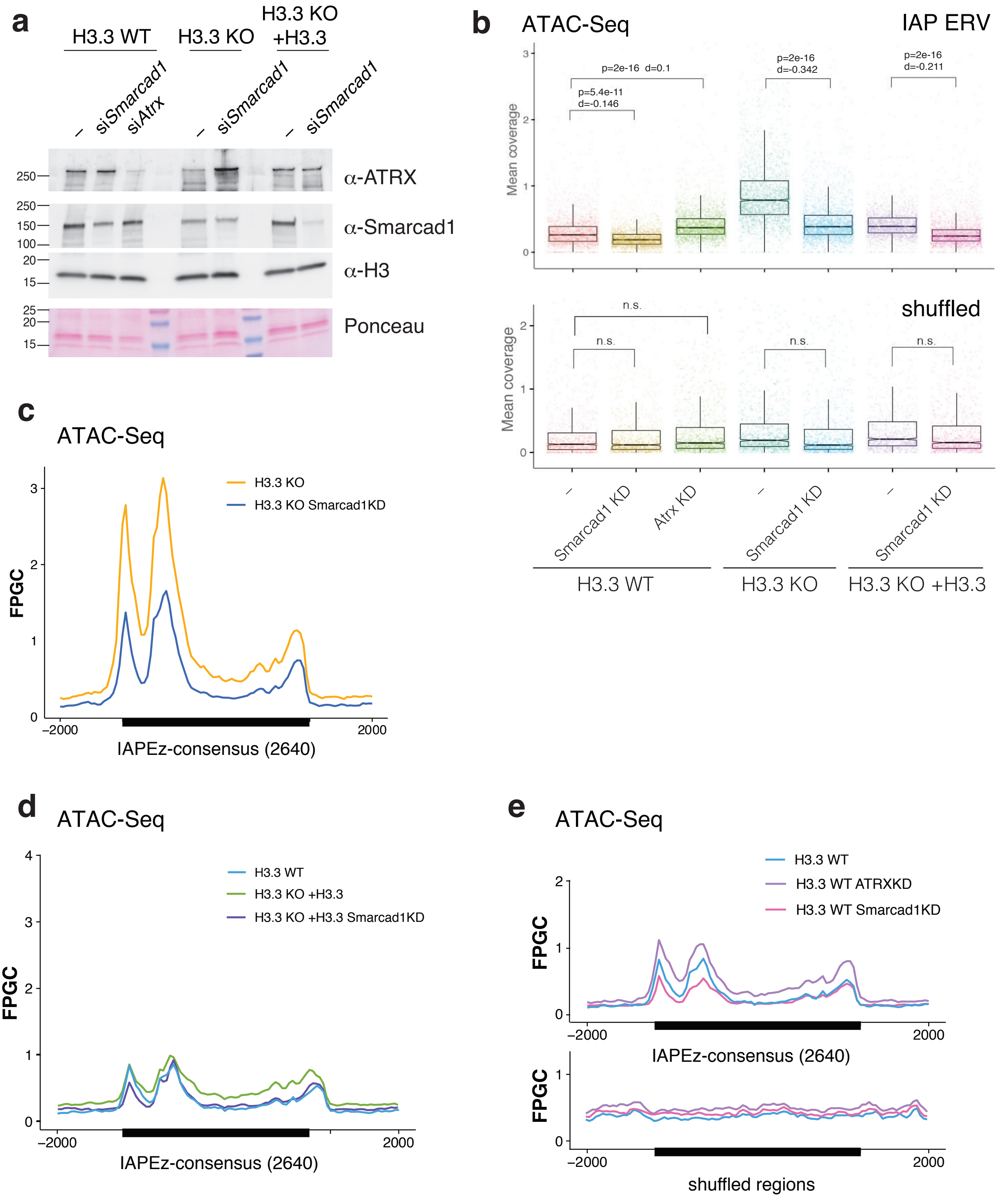

Supplementary Figure 12 - related to Figure 5e,f,g - Replicate ATAC-Seq experiment with additional knockdown of ATRX and additional cell lines

e Western blot showing Smarcad1 and Atrx siRNA treatment of H3.3 WT and H3.3 KO cell lines, as well as H3.3KO cell line rescued with H3.3 (see Figure 4a). **b** Boxplots showing H3.3 and ATAC-Seq signal for the conditions above at 2640 shared IAP ERVs (top) or 2640 randomized control regions (bottom). Tukey-style center (median), box (first and third quartiles) and whiskers (the maximal 1.5x IQR range). Two-sided Paired T test p value and Cohen's effect size d are given for each pairwise comparison. Data shown from n=1 biological replicate. Source data are provided as a Source Data file. **c** ATAC-Seq average coverage (FPRC) over 2640 shared IAP ERVs for H3.3 KO cell line untreated or treated with Smarcad1 siRNA. **d** ATAC-Seq average coverage (FPRC) over 2640 shared IAP ERVs for H3.3 WT and H3.3 KO +H3.3 rescue cell line untreated or treated with Smarcad1 siRNA. **e** ATAC-Seq average coverage (FPRC) over 2640 shared IAP ERVs for H3.3 WT cell line untreated or treated with Smarcad1 or ATRX siRNA (top). ATAC-Seq average coverage (FPRC) over 2640 random control regions for H3.3 WT cell line untreated or treated with Smarcad1 or ATRX siRNA (bottom).

Supplementary Figure 13

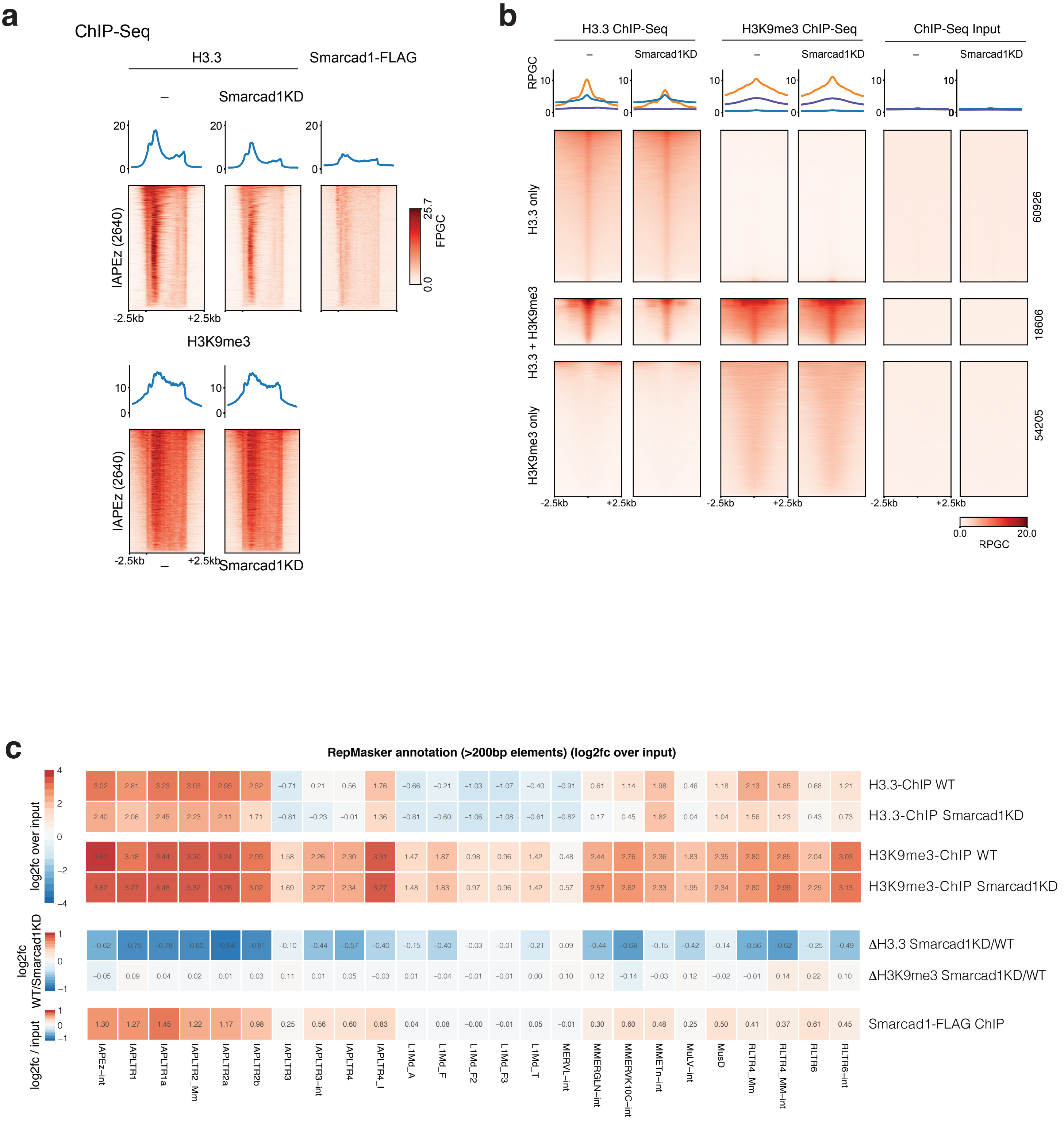

Supplementary Figure 13 - related to Figure 6b,d,e - Smarcad1 knockdown reduces heterochromatic H3.3 enrichment

a Density heatmaps and average profiles of H3.3, H3K9me3 and Smarcad1-FLAG [12] ChIP-Seq signal over 2640 shared IAP ERVs and flanking regions, in H3.3 WT cells with or without Smarcad1 knockdown. **b** ChIP-Seq density heatmaps and average profiles of H3.3 and H3K9me3 in H3.3 WT mESC with or without 48h Smarcad1 knockdown over H3.3-only (same as Figure 6d), H3K9me3-only and H3.3+H3K9me3 peaks [6]. **c** Mean ChIP-Seq read density heatmap showing enrichments of Smarcad1-FLAG [12], H3K9me3 and H3.3 over major ERV and LINE repeat families as annotated by RepeatMasker. Only instances larger than 200bp were included in each analysis and the log2-fold enrichment of each ChIP over its respective Input are given. Further, the pairwise log2fold ratio between WT and Smarcad1 KD for H3.3 and H3K9me3 were plotted (middle). Source data are provided as a Source Data file.

Supplementary Figure 14

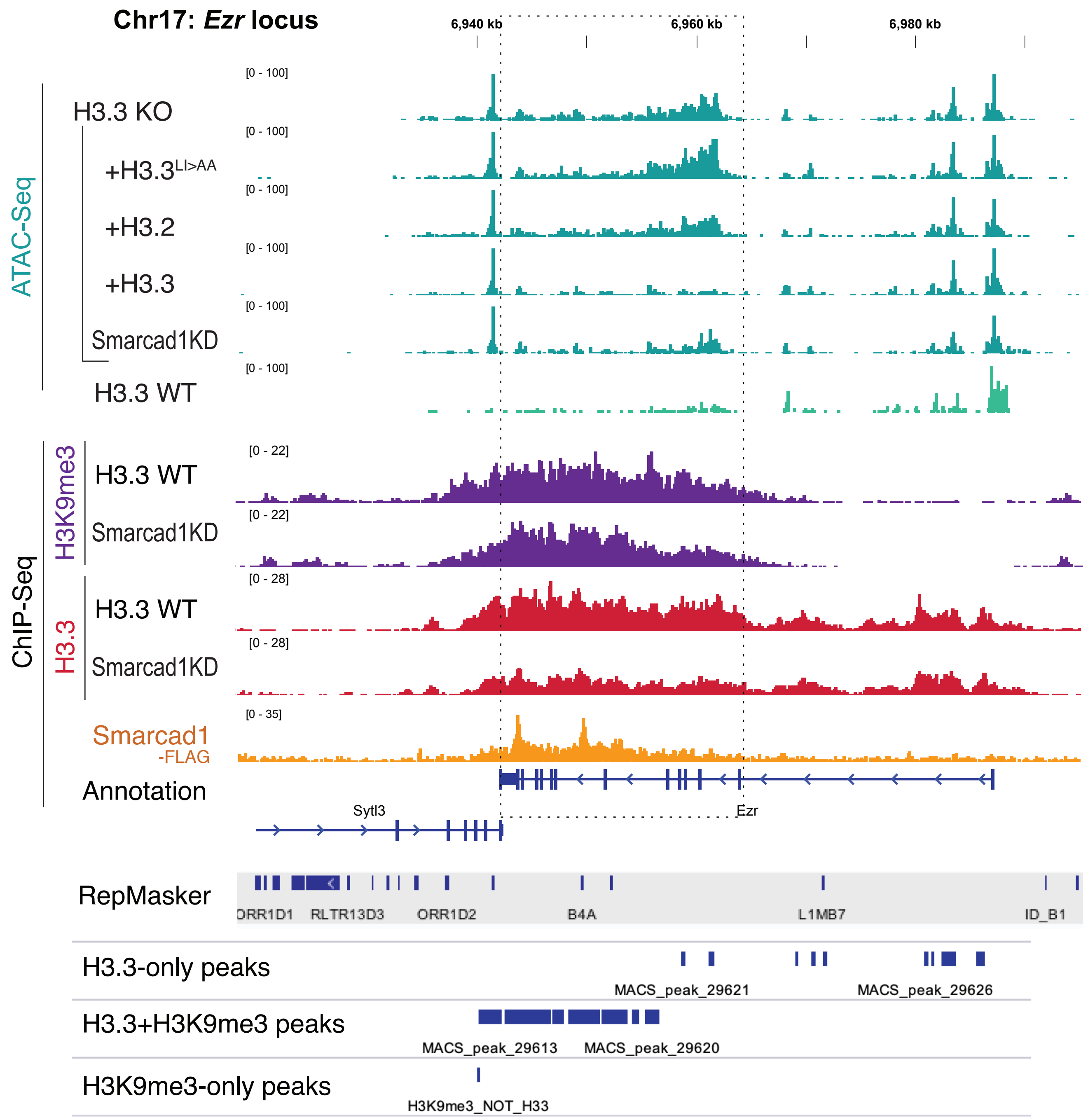

Supplementary Figure 14 - related to Figures 5a and 6d - Example of dynamic non-repetitive interstitial heterochromatin at *Ezr* locus

Genome tracks of ATAC-Seq, H3K9me3, H3.3, Smarcad1 [12] ChIP-Seq in indicated conditions. All tracks were normalized to Reads Per Genomic Content (RPGC).

Supplementary Figure 15

Consensus set of 2640 IAP ERVs common ERVs accross datasets from different mouse backgrounds

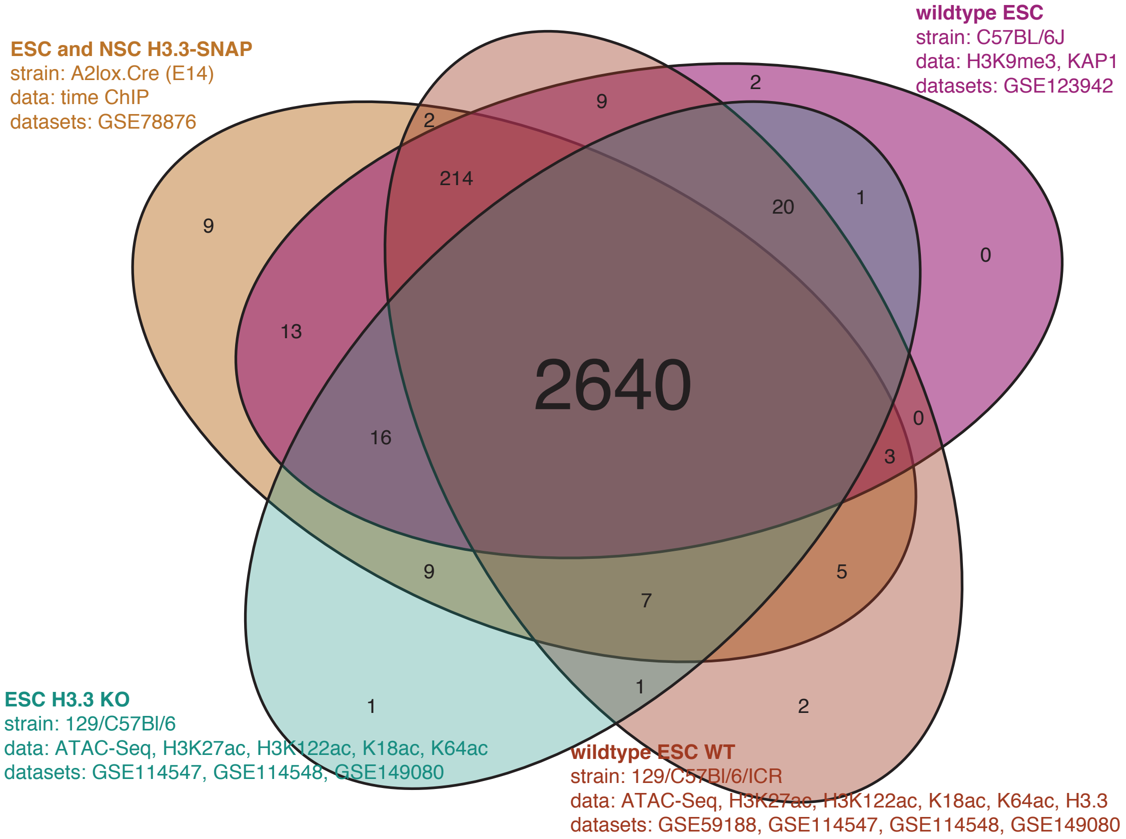

Supplementary Figure 15 - Consensus IAP ERV elements shared across datasets from different mouse strains. Venn diagram showing overlap between the datasets used to collect evidence from shared IAP ERV elements on a per-locus basis. Since evidence was based on paired-end reads anchored in the unique flanking region (see Methods), we could only include paired-end datasets in the census. For each individual study, a IAP ERV element was retained if it showed evidence on any of the datasets. From a total set of 3046 full-length IAP ERVs obtained from UCSC RepMasker track, a curated consensus final dataset of 2640 loci was obtained.

Supplementary Table 1: Oligonucleotides

Oligonucleotides were ordered desalted from IDT. Sequences are given below.

Name	Sequence
Tn5ME-A (Illumina FC-121-1030)	TCGTCGGCAGCGTCAGATGTGTATAAGAGACAG
Tn5ME-B (Illumina FC-121-1031)	GTCTCGTGGGCTCGGAGATGTGTATAAGAGACAG
Tn5MErev	pCTGTCTCTTATACACATCT
v2_Ad1.1_TAGATCGC	AATGATACGGCGACCACCGAGATCTACACTAGATCGCTCGTCGGCAGCGTCAGATGTGTAT
v2_Ad1.2_CTCTCTAT	AATGATACGGCGACCACCGAGATCTACACCTCTCTATTCGTCGGCAGCGTCAGATGTGTAT
v2_Ad1.3_TATCCTCT	AATGATACGGCGACCACCGAGATCTACACTATCCTCTTCGTCGGCAGCGTCAGATGTGTAT
v2_Ad1.4_AGAGTAGA	AATGATACGGCGACCACCGAGATCTACACAGAGTAGATCGTCGGCAGCGTCAGATGTGTAT
v2_Ad1.5_GTAAGGAG	AATGATACGGCGACCACCGAGATCTACACGTAAGGAGTCGTCGGCAGCGTCAGATGTGTAT
v2_Ad1.6_ACTGCATA	AATGATACGGCGACCACCGAGATCTACACACTGCATATCGTCGGCAGCGTCAGATGTGTAT
v2_Ad1.7_AAGGAGTA	AATGATACGGCGACCACCGAGATCTACACAAGGAGTATCGTCGGCAGCGTCAGATGTGTAT
v2_Ad1.8_CTAAGCCT	AATGATACGGCGACCACCGAGATCTACACCTAAGCCTTCGTCGGCAGCGTCAGATGTGTAT
v2_Ad1.9_TGGAAATC	AATGATACGGCGACCACCGAGATCTACACTGGAAATCTCGTCGGCAGCGTCAGATGTGTAT
v2_Ad1.10_AACATGAT	AATGATACGGCGACCACCGAGATCTACACAACATGATTCGTCGGCAGCGTCAGATGTGTAT
v2_Ad1.11_TGATGAAA	AATGATACGGCGACCACCGAGATCTACACTGATGAAATCGTCGGCAGCGTCAGATGTGTAT
v2_Ad1.12_GTCGGACT	AATGATACGGCGACCACCGAGATCTACACGTCGGACTTCGTCGGCAGCGTCAGATGTGTAT
v2_Ad2.1_TAAGGCGA	CAAGCAGAAGACGGCATACGAGATTCGCCTTAGTCTCGTGGGCTCGGAGATGTG
v2_Ad2.2_CGTACTAG	CAAGCAGAAGACGGCATACGAGATCTAGTACGGTCTCGTGGGCTCGGAGATGTG

v2_Ad2.3_AGGCAGAA	CAAGCAGAAGACGGCATACGAGATTTCTGCCTGTCTCGTGGGCTCGGAGATGTG
v2_Ad2.4_TCCTGAGC	CAAGCAGAAGACGGCATACGAGATGCTCAGGAGTCTCGTGGGCTCGGAGATGTG
v2_Ad2.5_GGACTCCT	CAAGCAGAAGACGGCATACGAGATAGGAGTCCGTCTCGTGGGCTCGGAGATGTG
v2_Ad2.6_TAGGCATG	CAAGCAGAAGACGGCATACGAGATCATGCCTAGTCTCGTGGGCTCGGAGATGTG
v2_Ad2.7_CTCTCTAC	CAAGCAGAAGACGGCATACGAGATGTAGAGAGGTCTCGTGGGCTCGGAGATGTG
v2_Ad2.8_CAGAGAGG	CAAGCAGAAGACGGCATACGAGATCCTCTCTGGTCTCGTGGGCTCGGAGATGTG
v2_Ad2.9_GCTACGCT	CAAGCAGAAGACGGCATACGAGATAGCGTAGCGTCTCGTGGGCTCGGAGATGTG
v2_Ad2.10_CGAGGCTG	CAAGCAGAAGACGGCATACGAGATCAGCCTCGGTCTCGTGGGCTCGGAGATGTG
v2_Ad2.11_AAGAGGCA	CAAGCAGAAGACGGCATACGAGATTGCCTCTTGTCTCGTGGGCTCGGAGATGTG
v2_Ad2.12_GTAGAGGA	CAAGCAGAAGACGGCATACGAGATTCCTCTACGTCTCGTGGGCTCGGAGATGTG

Supplementary References

1. Yildirim, O. *et al.* A system for genome-wide histone variant dynamics in ES cells reveals dynamic MacroH2A2 replacement at promoters. *PLoS Genet.* **10,** e1004515 (2014).
2. Deaton, A. M. *et al.* Enhancer regions show high histone H3.3 turnover that changes during differentiation. *elife* **5,** (2016).
3. Shi, H. *et al.* ZFP57 regulation of transposable elements and gene expression within and beyond imprinted domains. *Epigenetics Chromatin* **12,** 49 (2019).
4. Martire, S. *et al.* Phosphorylation of histone H3.3 at serine 31 promotes p300 activity and enhancer acetylation. *Nat. Genet.* **51,** 941–946 (2019).
5. Bunch, H. *et al.* TRIM28 regulates RNA polymerase II promoter-proximal pausing and pause release. *Nat. Struct. Mol. Biol.* **21,** 876–883 (2014).
6. Elsässer, S. J., Noh, K.-M., Diaz, N., Allis, C. D. & Banaszynski, L. A. Histone H3.3 is required for endogenous retroviral element silencing in embryonic stem cells. *Nature* **522,** 240–244 (2015).
7. Bogu, G. K. *et al.* Chromatin and RNA maps reveal regulatory long noncoding rnas in mouse. *Mol. Cell. Biol.* **36,** 809–819 (2015).
8. Chronis, C. *et al.* Cooperative binding of transcription factors orchestrates reprogramming. *Cell* **168,** 442-459.e20 (2017).
9. Schlesinger, S. *et al.* A hyperdynamic H3.3 nucleosome marks promoter regions in pluripotent embryonic stem cells. *Nucleic Acids Res.* **45,** 12181–12194 (2017).
10. Ha, M., Kraushaar, D. C. & Zhao, K. Genome-wide analysis of H3.3 dissociation reveals high nucleosome turnover at distal regulatory regions of embryonic stem cells. *Epigenetics Chromatin* **7,** 38 (2014).
11. Ostapcuk, V. *et al.* Activity-dependent neuroprotective protein recruits HP1 and CHD4 to

control lineage-specifying genes. *Nature* **557,** 739–743 (2018).

12. Sachs, P. *et al.* SMARCAD1 ATPase activity is required to silence endogenous retroviruses in embryonic stem cells. *Nat. Commun.* **10,** 1335 (2019).

Figure 4a uncropped images

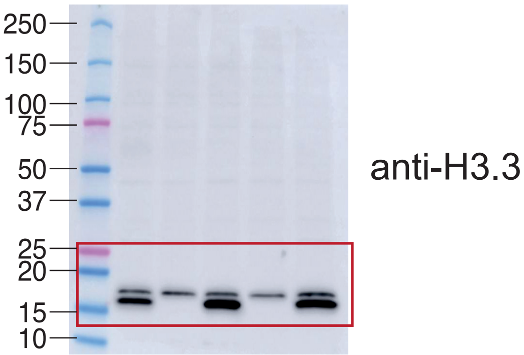

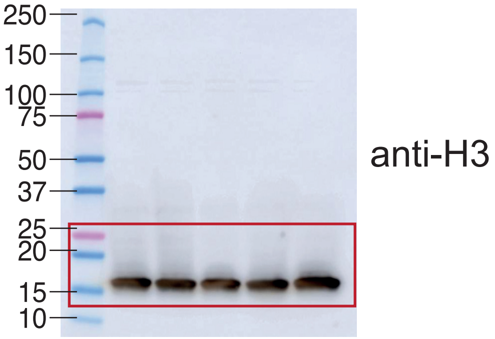

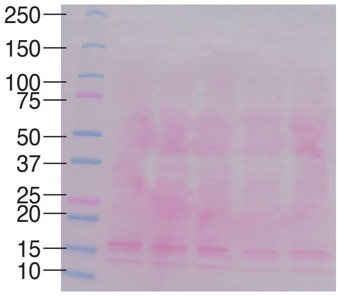

Uncropped gel images Figure 5e

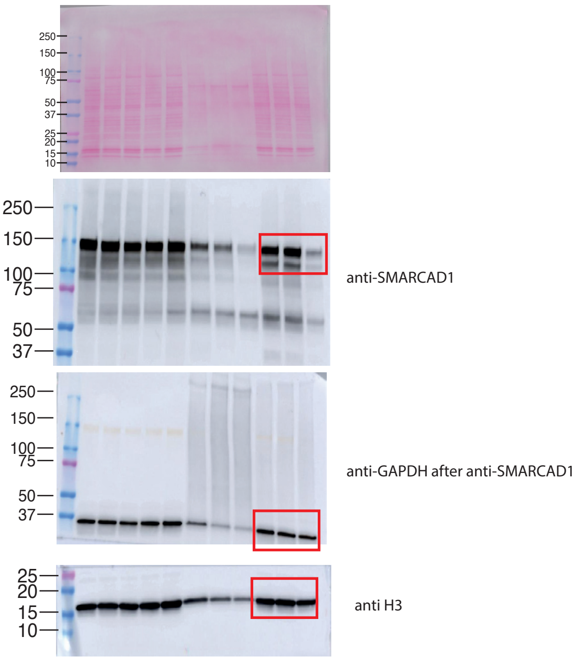

Uncropped gel images Supplementary Figure 12

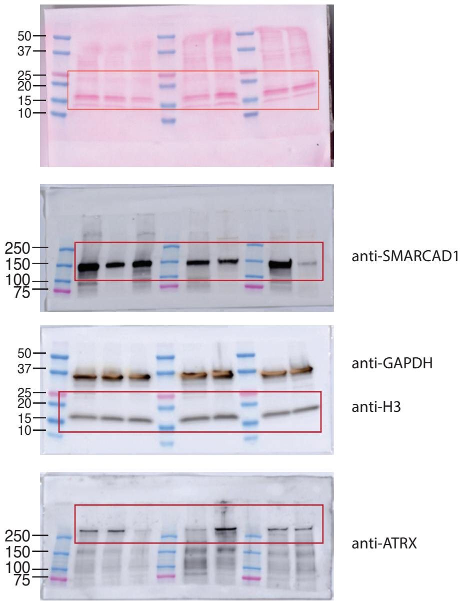

Supplement: Supplementary file 1 — Supplementary Information [file 41467_2020_18863_MOESM1_ESM.pdf]
