# Supplementary material for: An embryonic stem cell-specific heterochromatin state promotes core histone exchange in the absence of DNA accessibility
Source: Nat Commun. 2020 Oct 9;11:5095. doi: 10.1038/s41467-020-18863-1 (PMC7547087; doi:10.1038/s41467-020-18863-1)
Supplement: Supplementary file 3 — Description of Additional Supplementary Files [file 41467_2020_18863_MOESM3_ESM.pdf]

Description of Additional Supplementary Files

File Name: Supplementary Data 1
Description: A list of published datasets and accession numbers used in this study.
