# Supplementary material for: An embryonic stem cell-specific heterochromatin state promotes core histone exchange in the absence of DNA accessibility
Source: Nat Commun. 2020 Oct 9;11:5095. doi: 10.1038/s41467-020-18863-1 (PMC7547087; doi:10.1038/s41467-020-18863-1)
Supplement: Supplementary file 5 — Reporting Summary [file 41467_2020_18863_MOESM5_ESM.pdf]

nature research

Corresponding author(s): Simon Elsässer

Last updated by author(s): May 16, 2020

Reporting Summary

Nature Research wishes to improve the reproducibility of the work that we publish. This form provides structure for consistency and transparency in reporting. For further information on Nature Research policies, see Authors & Referees and the Editorial Policy Checklist.

Statistics

For all statistical analyses, confirm that the following items are present in the figure legend, table legend, main text, or Methods section.

n/a	Confirmed	
☐	☒	The exact sample size (*n*) for each experimental group/condition, given as a discrete number and unit of measurement
☒	☐	A statement on whether measurements were taken from distinct samples or whether the same sample was measured repeatedly
☐	☒	The statistical test(s) used AND whether they are one- or two-sided<br>*Only common tests should be described solely by name; describe more complex techniques in the Methods section.*
☒	☐	A description of all covariates tested
☒	☐	A description of any assumptions or corrections, such as tests of normality and adjustment for multiple comparisons
☐	☒	A full description of the statistical parameters including central tendency (e.g. means) or other basic estimates (e.g. regression coefficient) AND variation (e.g. standard deviation) or associated estimates of uncertainty (e.g. confidence intervals)
☐	☒	For null hypothesis testing, the test statistic (e.g. *F*, *t*, *r*) with confidence intervals, effect sizes, degrees of freedom and *P* value noted<br>*Give P values as exact values whenever suitable.*
☒	☐	For Bayesian analysis, information on the choice of priors and Markov chain Monte Carlo settings
☒	☐	For hierarchical and complex designs, identification of the appropriate level for tests and full reporting of outcomes
☐	☒	Estimates of effect sizes (e.g. Cohen's *d*, Pearson's *r*), indicating how they were calculated

Our web collection on statistics for biologists contains articles on many of the points above.

Software and code

Policy information about availability of computer code

Data collection	Illumina's Bcl2fastq (v2.20) was used to demultiplex NextSeq 500 output to primary FASTQ
Data analysis	A NextFlow (v20.07.1) pipeline configuration and additional scripts to process the data is provided on GitHub (https://github.com/elsasserlab/publicchip). Public tools used include bowtie2 (v 2.3.5.1), deeptools (v 3.1.0), samtools (v1.10), picard (v 2.20.4), bedtools (v2.27.1), MACS2 (v 2.1.2), IGV (2.3), matplotlib (v 3.1.1), rtracklayer (v1.48.0), pheatmap (v1.0.12) . Further information including commands parameters used are provided in Methods section.

For manuscripts utilizing custom algorithms or software that are central to the research but not yet described in published literature, software must be made available to editors/reviewers. We strongly encourage code deposition in a community repository (e.g. GitHub). See the Nature Research guidelines for submitting code & software for further information.

Data

Policy information about availability of data

All manuscripts must include a data availability statement. This statement should provide the following information, where applicable:

- Accession codes, unique identifiers, or web links for publicly available datasets
- A list of figures that have associated raw data
- A description of any restrictions on data availability

Source data underlying figures 1a, 1e, 2d, 3c, 4a, 4b, 5e, 5f, 6c, 6e, 6f, extended 12b and extended 13c are provided as a Source Data file.
ATAC-Seq and ChIP-Seq data have been deposited at Gene Expression Omnibus under GSE149080 [https://www.ncbi.nlm.nih.gov/geo/query/acc.cgi?acc=GSE149080].
Data sources from publications analysed in this study are listed in Supplementary Table 1. Most of the downstream analyses come from bigWig files that can be obtained by running the Nextflow pipeline provided as part of the code repository attached to this publication. These bigWig files are also available upon request to

the authors. Various metadata, genomic annotations used throughout the analyses, IGV sessions are available at the code repository and Mendeley Data rk28yn8gwg [http://dx.doi.org/10.17632/rk28yn8gwg.1].

Field-specific reporting

Please select the one below that is the best fit for your research. If you are not sure, read the appropriate sections before making your selection.

☒ Life sciences ☐ Behavioural & social sciences ☐ Ecological, evolutionary & environmental sciences

For a reference copy of the document with all sections, see nature.com/documents/nr-reporting-summary-flat.pdf

Life sciences study design

All studies must disclose on these points even when the disclosure is negative.

Sample size	For nucleosome dynamics in mouse ESC, five independent datasets were analyzed and results for all datasets are shown in main and extended data figures. One dataset contained biological triplicates, the remaining datasets are time series with n=1 for each time point. For ATAC-Seq, one published dataset was reanalyzed and two new dataset (independent biological and technical replicates) were generated containing matching conditions to the published dataset. For ChIP-Seq, a single replicate comparison was acquired.
Data exclusions	All existing datasets for H3.3 dynamics were represented in this study and taken into account for the conclusions. Where available, e.g. for H3K9me3 and ATAC-Seq, several datasets from different studies were analyzed. While only one dataset each was chosen to be represented in the figures, we analyzed all remaining replicates and ensured that all datasets agreed on the conclusions drawn. No experimental data was excluded. In one experiment, siRNA knockdown efficiency was too low, thus we did not proceed to collect ATAC-Seq/ChIP-Seq data and instead experiment was repeated from the start.
Replication	Key observations regarding nucleosome dynamics were replicated in multiple datasets, using sifferent types of pulse/chase experimental setups, mouse ESC lines, ChIP protocols and antibodies, and were performed and published by different laboratories. Key conclusions from ATAC-Seq experiments are replicated in our two and a third published datasets.
Randomization	No randomization was done since the data was processed though a standardized analysis that excludes human bias
Blinding	No blinding was done since the data was processed though a standardized analysis that excludes human bias

Reporting for specific materials, systems and methods

We require information from authors about some types of materials, experimental systems and methods used in many studies. Here, indicate whether each material, system or method listed is relevant to your study. If you are not sure if a list item applies to your research, read the appropriate section before selecting a response.

Materials & experimental systems

n/a	Involved in the study	
☐	☒	Antibodies
☐	☒	Eukaryotic cell lines
☒	☐	Palaeontology
☒	☐	Animals and other organisms
☒	☐	Human research participants
☒	☐	Clinical data

Methods

n/a	Involved in the study	
☐	☒	ChIP-seq
☒	☐	Flow cytometry
☒	☐	MRI-based neuroimaging

Antibodies

Antibodies used	For ChIP, a-H3.3 (Millipore, 09-838, Lot 3273632) and a-H3K9me3 (Abcam, ab8898, Lot GR3244172-2) were used. For western blot, 1:200 a-ATRX (Santa Cruz, sc-15408, Lot #A0915), 1:5000 a-H3 (Activemotif, 39763, Lot 20418023), 1:500 a-H3.3 (Millipore, 09-838 Lot 3273632), 1:5000 a-GAPDH (Millipore, AB2302, Lot 2967896), 1:2000 a-SMARCAD1 (Sigma, HPA016737, Lot 000004262). Secondary antibodies: 1:5000 a-Chicken-HRP (Invitrogen, A16054, Lot 58-39-081517), 1:5000 a-Mouse-HRP (BioRad, 1721011), 1:5000 a-Rabbit-HRP (BioRad, 172101) were used
Validation	a-H3.3 (Millipore, 09-838, Lot 3273632): validation data provided by the supplier (https://www.merckmillipore.com/SE/en/product/Anti-Histone-H3.3-Antibody,MM_NF-09-838) and also characterized in detail in Banaszynski et. al., Cell, 2013.<br>a-H3K9me3 (Abcam, ab8898, Lot GR3244172-2): validation data provided by the supplier (https://www.abcam.com/histone-h3-tri-methyl-k9-antibody-chip-grade-ab8898.html)<br>a-ATRX (Santa Cruz, sc-15408, Lot #A0915) was validated by knockdown in our study and independently confirmed by lack of reactivity with ATRX KO cells available in our laboratory.<br>a-H3 (Activemotif, 39763, Lot 20418023) validation data provided by the supplier (https://www.activemotif.com/catalog/details/39763/histone-h3-antibody-clone-mabi-0301)

1:5000 a-GAPDH (Millipore, AB2302, Lot 2967896) validation data provided by the supplier (https://www.merckmillipore.com/SE/en/product/Anti-GAPDH-Antibody,MM_NF-AB2302)
a-SMARCAD1 (Sigma, HPA016737, Lot 000004262) validation data provided by the supplier for human (https://www.atlasantibodies.com/products/antibodies/primary-antibodies/triple-a-polyclonals/smarcad1-antibody-hpa016737/) and validated in our lab by siRNA knockdown

Eukaryotic cell lines

Policy information about cell lines

Cell line source(s)	H3.3 knockout (H3.3KO) and wildtype control (H3.3WT) cells were acquired from Laura Banaszynski/David Allis Laboratory (Banasczynski et. al., Cell, 2013)
Authentication	After acquisition, the cell line has not been further authenticated.
Mycoplasma contamination	All cell lines have been tested negative for Mycoplasma contamination
Commonly misidentified lines (See ICLAC register)	*Name any commonly misidentified cell lines used in the study and provide a rationale for their use.*

ChIP-seq

Data deposition

☒ Confirm that both raw and final processed data have been deposited in a public database such as GEO.

☒ Confirm that you have deposited or provided access to graph files (e.g. BED files) for the called peaks.

Data access links *May remain private before publication.*	GSE149080 [https://www.ncbi.nlm.nih.gov/geo/query/acc.cgi?acc=GSE149080]<br>Mendeley Data rk28yn8gwg [http://dx.doi.org/10.17632/rk28yn8gwg.1]<br>https://github.com/elsasserlab/publicchip.
Files in database submission	12 ATAC-Seq datasets, 6 ChIP-Seq datasets
Genome browser session (e.g. UCSC)	https://export.uppmax.uu.se/snic2020-6-3/Navarro/igv/

Methodology

Replicates	Key observations regarding nucleosome dynamics were replicated in multiple datasets, using different types of pulse/chase experimental setups, mouse ESC lines, ChIP protocols and antibodies, and were performed and published by different laboratories. ATAC-Seq data provided in two independent biological and technical replicates and replicated also inone published dataset. H3.3 and H3K9me3 ChIP were generated in single experimental conditions.
Sequencing depth	Sequencing depth was between 10 and 50 Mio paired-end reads for ATAC-Seq. 40-100 Mio paired-end reads for ChIP-Seq
Antibodies	Antibody sources and validation above
Peak calling parameters	MACS2 broadPeak, default --broad-cutoff 0.05
Data quality	Data quality of the published datasets has been assessed with FastQC
Software	A NextFlow pipeline configuration and additional scripts to process the data is provided on GitHub (https://github.com/elsasserlab/publicchip).
