# Supplementary material for: An embryonic stem cell-specific heterochromatin state promotes core histone exchange in the absence of DNA accessibility
Source: Nat Commun. 2020 Oct 9;11:5095. doi: 10.1038/s41467-020-18863-1 (PMC7547087; doi:10.1038/s41467-020-18863-1)
Supplement: Supplementary file 6 — Source Data [file 41467_2020_18863_MOESM6_ESM.zip › source_data/fig5.zip!/fig5/fig05_e/5e.pdf]

Uncropped gel images Figure 5e

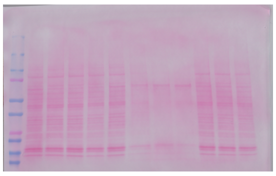

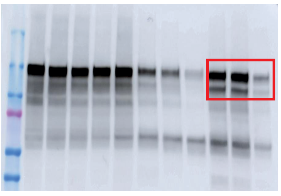

anti-SMARCAD1

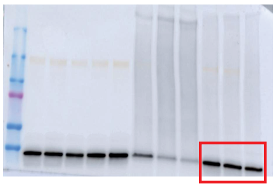

anti-GAPDH after anti-SMARCAD1

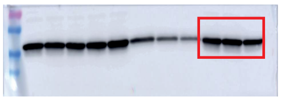

anti H3
