# Supplementary material for: An embryonic stem cell-specific heterochromatin state promotes core histone exchange in the absence of DNA accessibility
Source: Nat Commun. 2020 Oct 9;11:5095. doi: 10.1038/s41467-020-18863-1 (PMC7547087; doi:10.1038/s41467-020-18863-1)

Figure 4a uncropped images

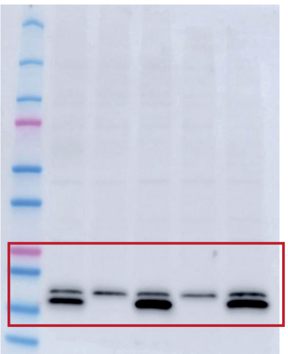

anti-H3.3

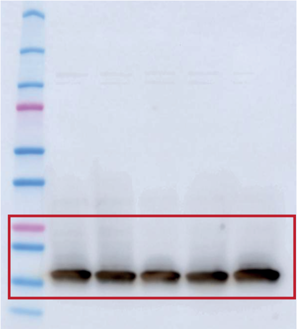

anti-H3

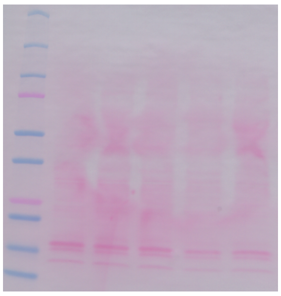

Supplement: Supplementary file 6 — Source Data [file 41467_2020_18863_MOESM6_ESM.zip › source_data/fig4.zip!/fig4/fig04_a/fig4a.pdf]
